# UHRF1 overexpression is involved in cell proliferation and biochemical recurrence in prostate cancer after radical prostatectomy

**DOI:** 10.1186/s13046-016-0308-0

**Published:** 2016-02-17

**Authors:** Xuechao Wan, Shu Yang, Wenhua Huang, Denglong Wu, Hongbing Chen, Ming Wu, Junliang Li, Tao Li, Yao Li

**Affiliations:** Department of Urology, Tongji Hospital, Tongji University School of Medicine, Shanghai, 200065 People’s Republic of China; State Key Laboratory of Genetic Engineering, Institute of Genetics, School of Life Science, Fudan University, Shanghai, 200433 People’s Republic of China; Department of Urology, Hefei First People’s Hospital, Hefei, Anhui 230061 People’s Republic of China; Shanghai Institute of Planned Parenthood Research Hospital, WHO Collaborating Center for Research in Human Reproduction, Shanghai, People’s Republic of China

**Keywords:** Prostate cancer (PCa), *UHRF1*, Biomarker, Radical prostatectomy (RP), Biochemical recurrence (BCR)

## Abstract

**Background:**

Biochemical recurrence (BCR) is widely used to define the treatment success and to make decisions on if or how to initiate a secondary therapy, but uniform criteria to define BCR after radical prostatectomy (RP) is not yet completely assessed. UHRF1 has a unique function in regulating the epigenome by linking DNA methylation with histone marks. The clinical value of UHRF1 in PCa has not been well done. Therefore, we evaluated the prognostic significance of UHRF1.

**Method:**

UHRF1 expression in PCa cells was monitored by qRT-PCR and Western blot analyses. UHRF1 expression was knocked down using specific siRNAs, and the effects of knockdown on the proliferation, migration, cell cycle, and apoptosis of PCa cell lines were investigated. UHRF1 protein expression was evaluated in 225 PCa specimens using immunohistochemistry in tissue microarrays. Correlations between UHRF1 expression and the clinical features of PCa were assessed.

**Results:**

The results showed that UHRF1 was overexpressed in almost all of the PCa cell lines. In PCa cells, UHRF1 knockdown inhibited cell proliferation and migration, and induced apoptosis. UHRF1 expression levels were correlated with some clinical features of PCa. Multivariate analysis showed that UHRF1 expression was an independent prognostic factor for biochemical recurrence-free survival.

**Conclusions:**

UHRF1 functions as an oncogene in prostate cancer and appears to be capable of predicting the risk of biochemical recurrence in PCa patients after radical prostatectomy, and may serve as a potential therapeutic target for PCa.

## Background

Prostate cancer (PCa) is the most common genitourinary malignancy in males worldwide. Since the advent of prostate-specific antigen (PSA) testing in the late 1980s, different diagnostic and treatment approaches for PCa have been developed [1]. Currently, radical prostatectomy (RP) is the primary treatment for localized PCa [2]. However, during long-term follow-up after RP, about 25–50 % of patients develop biochemical recurrence (BCR), which is generally the earliest indicator of recurrent disease [3]. BCR is widely used to define the treatment success and to make decisions on if or how to initiate a secondary therapy, but uniform criteria to define BCR after radical prostatectomy (RP) is not yet completely assessed. EAU guidelines define a PSA cutoff post-RP of ≥0.2 ng/ml [4]. Although at least 10 definitions of BCR have been proposed. PSA levels ≥4 ng/ml have been shown to have the highest correlation with the risk of clinical progression, which tightly associates with metastatic disease progression (MP) and prostate cancer–specific mortality (PCSM) [5]. Furthermore, PSA kinetics is relevant for the BRC definition. EAU guidelines define BCR when two sequential PSA values post-RP are ≥0.2 ng/ml. CAU (China Urological Association) guidelines define BCR in agreement with that of EAU so BCR in this study is defined as two sequential PSA values post-RP ≥ 0.2 ng/ml. Patients with an equivalent PSA level, Gleason score, and pathological stage can have different clinical outcomes for PCa, depending on which molecular heterogeneous subtypes is involved [6]. Therefore, developing a better understanding of the molecular pathology of PCa is critical for establishing effective indicators of prognosis and therapeutic strategies against this potentially fatal disease.

Ubiquitin-like, containing PHD ring finger 1 (UHRF1, also known as ICBP90) was originally identified as a protein whose subcellular expression pattern coincided with sites of DNA replication [7, 8]. UHRF1 is characterized by a SET and ring-associated (SRA) domain, which is found only in the UHRF family. UHRF1 transmits the DNA methylation status from mother to daughter cells by recognizing hemimethylated DNA, recruiting DNA methyltransferase 1, and proliferating cell nuclear antigen to the site to methylate newly synthesized DNA strands [9–12]. UHRF1 also binds histone H3 that is trimethylated at lysine 4 and 9 (H3K4me3 and H3K9me3), and UHRF1 plays a role in maintaining this histone modification in heterochromatin [13, 14]. Thus, UHRF1 has a unique function in regulating the epigenome by linking DNA methylation with histone marks [15, 16].

Previously, we showed that the H3 trimethylation status at lysine 4 can predict the risk of BCR in low-grade PCa (Gleason score ≤ 6) after RP [17]. Therefore, UHRF1 might play an important role in tumourigenesis in the prostate. Expression of UHRF1 is upregulated in several cancers (e.g., breast, lung, pancreatic, and cervical cancers) [18–21], suggesting that this protein is involved cancer development and progression. If so, then UHRF1 could be an important diagnostic biomarker and prognostic indicator for cancer. However, the expression and functional roles of UHRF1 in PCa remain unclear.

In this study, the expression of UHRF1 in PCa was analysed at the cell and tissue levels. The aims of this study were to investigate whether UHRF1 could be a novel diagnostic marker and used as a therapeutic target in PCa, to determine the underlying molecular mechanism, and to understand the clinical importance of UHRF1 in prostate carcinogenesis.

## Methods

### Patients

Tumour or tissue samples were extracted from patients with PCa (*n* = 225), prostatic intraepithelial neoplasia (PIN; *n* = 19), or normal prostates (*n* = 25). Normal prostate tissue was obtained from patients with bladder cancer after total cystectomy. PCa and PIN tissues were from patients with PCa who underwent RP and regional lymph node (LN) dissection at TongJi Hospital, a subsidiary of TongJi University, between January 2001 and December 2013. Patients did not receive any preoperative treatment. Histopathologic features of the tumour samples were classified according to the Gleason scoring system and the 2002 TNM classification system. The Research Ethics Committee of TongJi Hospital approved this protocol, and verbal consent was obtained from all patients.

### Prostate tissue microarray and immunohistochemical analyses

A prostate tissue microarray (TMA) was constructed using formalin-fixed, paraffin-embedded prostate tissue samples. Areas of invasive adenocarcinoma were identified according to the corresponding haematoxylin and eosin (H&E)-stained slides. Two replicate tumour samples (1 mm in diameter) were taken from the donor tissue blocks in a highly representative fashion and arrayed into a recipient paraffin block (35 mm × 622 mm × 65 mm) using a tissue microarrayer (Beecher Instrument Inc., Sun Prairie, WI), as previously described [22].

UHRF1 was detected with a specific rabbit polyclonal antibody (ab52039, Abcam Inc., Cambridge, MA), which was tested and optimized on whole-tissue sections and test arrays. Once an appropriate dilution (1:100) and incubation time had been determined, two TMA sections containing all of the patient samples were stained for UHRF1 by standard two-step immunohistochemistry. Immediately before staining, the TMA sections were cut with a sectioning aid (Instrumedics, St. Louis, MO), deparaffinised in xylene, and rehydrated in a graded alcohol series. Endogenous peroxidase was quenched with 0.3 % hydrogen peroxide in methanol at room temperature (24 °C). Sections were placed in a 120 °C, 0.01 M sodium citrate buffer (pH 6.0) for antigen retrieval. The Dako Envision System was used as the secondary antibody. Sections were visualized with diaminobenzidine (DAB), counterstained with haematoxylin, dehydrated, and mounted. Identical TMA sections stained without primary antibody served as negative controls.

Two blinded observers performed independent semiquantitative assessments of antibody staining on the TMA sections. Each TMAs spot was scanned to assign the scores. UHRF1 expression was estimated as the total UHRF1 immunostaining score, which was calculated as the sum of an extent of staining score and an intensity score. The extent of staining score reflects the fraction of positive staining cells (score 0, 0 %; score 1, 1–25 %; score 2, 26–50 %; score 3, 51–75 %; score 4, 76–100 %). The intensity score represents the staining intensity (score 0, no staining signal; score 1, weak positive signal; score 2, moderate positive signal; score 3, strong positive signal). The sum of the intensity and extent of staining scores were used as the final staining score (0–7) for UHRF1. This relatively simple, reproducible scoring method gives highly concordant results between independent evaluators and has been used in previous studies [23]. For the purpose of statistical evaluation, tumours with a final staining score of ≥ 3 were considered to be UHRF1-positive.

### Image acquisition and management

Digital images were captured using the Nikon DS-Ri1 ECLIPSE Series (Nikon, Tokyo, Japan) with a 10× objective. TMA images were managed using NIS Element D4.00 software (Nikon).

### Cell culture

The PCa cell line LNCaP was purchased from the American Type Culture Collection (Manassas, VA) and confirmed by short tandem repeat (STR) analysis. PCa cell lines 22RV1, DU145, and PC-3 were obtained from the Cell Bank of the Chinese Academy of Sciences (Shanghai, China) and were authenticated by mycoplasma detection, DNA fingerprinting, isozyme detection, and cell vitality detection. All experiments were carried out with each cell line prior to passage 30. Cell lines were grown in RPMI 1640 medium supplemented with 10 % FBS, 1 % penicillin/streptomycin, 1 % nonessential amino acids, and 1 % mg/mL sodium pyruvate, and cultured at 37 °C in 5 % CO_2_. All cell culture reagents were purchased from Life Technologies (Grand Island, NY).

### RNA interference

Cells were seeded in 12-well plates and transfected with siRNAs using Lipofectamine 2000 (Life Technologies), in accordance with the manufacturer’s instructions. All siRNA oligonucleotides were synthesized by GenePharma (Shanghai, China). The following siRNAs were used: siUHRF1-535: 5′-GCCAGAGUGA GUCAGACAAT T-3′ and 5′-UUGUCUGACU CACUCUGGCT T-3′; siUHRF1-1453: 5′-GCACCAAGGA AUGUACCAUT T-3′ and 5′-AUGGUACAUU CCUUGGUGCT T-3′; and a scrambled siRNA control: 5′-UUCUCCGAAC GUGUCACGUT T-3′ and 5′-ACGUGACACG UUCGGAGAAT T-3′.

### Plasmids and cell transfection

Human cDNA of UHRF1 was was provided by Dr. Jiahuai Han (Xiamen University). Full-length cDNA of UHRF1 was cloned into expression plasmid pcDNA3.1(+) (Invitrogen). Transfections were carried out using Lipofectamine 2000, in accordance with the manufacturer’s protocol. The day before transfection, cells were seeded in six-well plates. A 800-ng sample of pcDNA3.1(+) or pcDNA3.1(+)-UHRF1 plasmid in 250 μL Opti-MEM medium (Life Technologies) was mixed with 5 μL of Lipofectamine 2000 dissolved in 250 μL of the same medium and allowed to stand at room temperature for 20 min. The resulting transfection solution (500 μL) was added to each well, which already contained 1.5 mL of Opti-MEM. Four hours later, the media was replaced with 2 mL of fresh RPMI 1640 medium.

### Real-time reverse transcription PCR analysis

Total RNA was extracted from 22RV1, DU145, PC-3, and LNCaP cells with Trizol reagent (Invitrogen). Reverse transcription was performed with the PrimeScript RT reagent kit (TaKaRa, Tokyo, Japan), by following the manufacturer’s protocol. Real-time quantitative PCR (qPCR) was performed using an ABI Prism 7900HT (Applied Biosystems, Foster City, CA). Primers for UHRF1 were: forward, 5′-TTGGCCAGAG TGCAAATGGA AGATCCAGGA GCTGTTCCA-3′ and reverse, 5′-AAGAGGGTAT GGCCGTCCTT CGGCTGTTTC TTGATTTTTG TAA-3′. Primers for β-actin were: forward, 5′-CCTCTCCCAA GTCCACACAG TGACGCTGGG GCTGGCATTG-3′ and reverse, 5′-GGGCACGAAG GCTCATCATT GCTCTTGCTG GGGCTGGTGG-3′. Primers were synthesized by SangonSBS Gentech Biotech (Shanghai, China). To determine the UHRF1 expression levels, Ct values were normalized to β-actin as an internal control. The relative mRNA expression was calculated using the 2^-ΔΔCt^ method. Each sample was run in triplicate to ensure accuracy.

### Western blot analysis

Cells were harvested 48 h after siRNA transfection by using a 1× SDS lysis buffer (50 mM Tris-HCl [pH 6.8], 100 mM DTT, 2 % SDS, 0.1 % bromphenol blue, 10 % glycerol). The protein concentration was determined by the BCA Protein Assay (Thermo Fisher Scientific, Waltham, MA), in accordance with the manufacturer's instructions. Proteins were separated by SDS-PAGE on a 12 % (w/v) polyacrylamide gel and transferred to membranes. Standard Western blot analyses were performed to measure the expression of β-actin (Sigma-Aldrich, MO). Secondary antibodies were purchased from Sigma-Aldrich. Signal intensities of the Western blots were quantified with the Quantity One software package (Bio-Rad, CA).

### Cell proliferation assay

Cell proliferation analysis was performed with Cell Counting Kit-8 (CCK-8; Dojindo Laboratories, Kumamoto, Japan), in accordance with the manufacturer’s protocol. Briefly, 5000 cells were seeded into each well of a 96-well plate. Proliferation was assessed at 0, 24, 48, 72, 96, and 120 h. CCK-8 (10 μL) was added to the well being harvested at each time point. Cells were incubated with CCK-8 for 2 h at 37 °C, and the absorbance at 450 nm was measured with a Microplate Reader ELx808 (Bio-Tek Instruments, Winooski, VT). The absorbance at 630 nm was used as a reference. Each experiment was performed at least in triplicate.

### Cell cycle assay

At 48 h after transfection, cells were removed from the culture plates with trypsin and washed with PBS. Cells were incubated with PBS containing 0.03 % Triton X-100, 100 ng/mL RNase A, and 50 ng/mL propidium iodide (PI) for 15 min in the dark. The percentage of cells in different cell cycle phases was measured with a FACStar flow cytometer (Becton-Dickinson, San Jose, CA) and analysed with ModFit software (Verity Software House, Topsham, ME).

### Annexin V-FITC apoptosis detection

Cells were seeded into 6-well plates and cultured for 48 h. Cells were collected 48 h after transfection. The level of apoptosis was determined with the FITC Annexin V Apoptosis Detection Kit (Becton-Dickinson) on a FACSCalibur flow cytometer (Becton-Dickinson), in accordance with the manufacturer’s instructions.

### Cell migration assay

Cell migration assays were performed in Transwell plates (8-μm pore size, 6.5-mm diameter; Corning Life Sciences, Lowell, MA), in accordance with the manufacturer’s protocol. Briefly, 2 × 10^4^ cells in 0.1 mL medium (supplemented with 1 % FBS) were seeded into the upper chamber of a Transwell plate, with 0.7 mL of medium (supplemented with 10 % FBS) in the lower chamber. Plates were incubated in a humidified incubator at 37 °C and 5 % CO_2_. After 120 h, the chambers were removed, and a cotton swab was used to scrape the cells that had not migrated from the upper side of the chamber. Cells in the lower chamber were fixed with methanol for 10 min, stained with 5 % Giemsa solution for 5 min, and washed twice with PBS. Cells that migrated through the Matrigel were counted using a microscope. For this assay, cells were treated with one of three siRNAs: scrambled siRNA (negative control), siUHRF1-535, or siUHRF1-1453. Cell migration was detected in triplicate for each siRNA, and the experiment was repeated three times.

### Statistical analysis

Numerical data are expressed as the mean ± standard deviation (SD). All in vitro experiments were performed at least three times. BCR is defined as two sequential PSA values post-RP ≥ 0.2 ng/ml. The BCR-free survival time was regulated as the time from the date of surgery to BCR. Follow-up data were updated in December 2013. The probability of survival was estimated using the Kaplan-Meier method. The log-rank test was used to compare differences in survival times. To analyse the relationship between UHRF1 expression and the clinical characteristics of the tumours, the chi-squared test, *t*-test, Fisher’s Exact test, or Mann-Whitney U-test was used as appropriate. A Cox proportional hazards model was used to establish independent factor(s) that predicted survival. All tests were two-sided, and statistical significance was set at *p* < 0.05. Statistical analysis was performed using the SPSS software package, version 15.0 (SPSS Inc., Chicago, IL).

## Results

### Expression levels of UHRF1 mRNA and protein in PCa cell lines

**Fig. 1:**
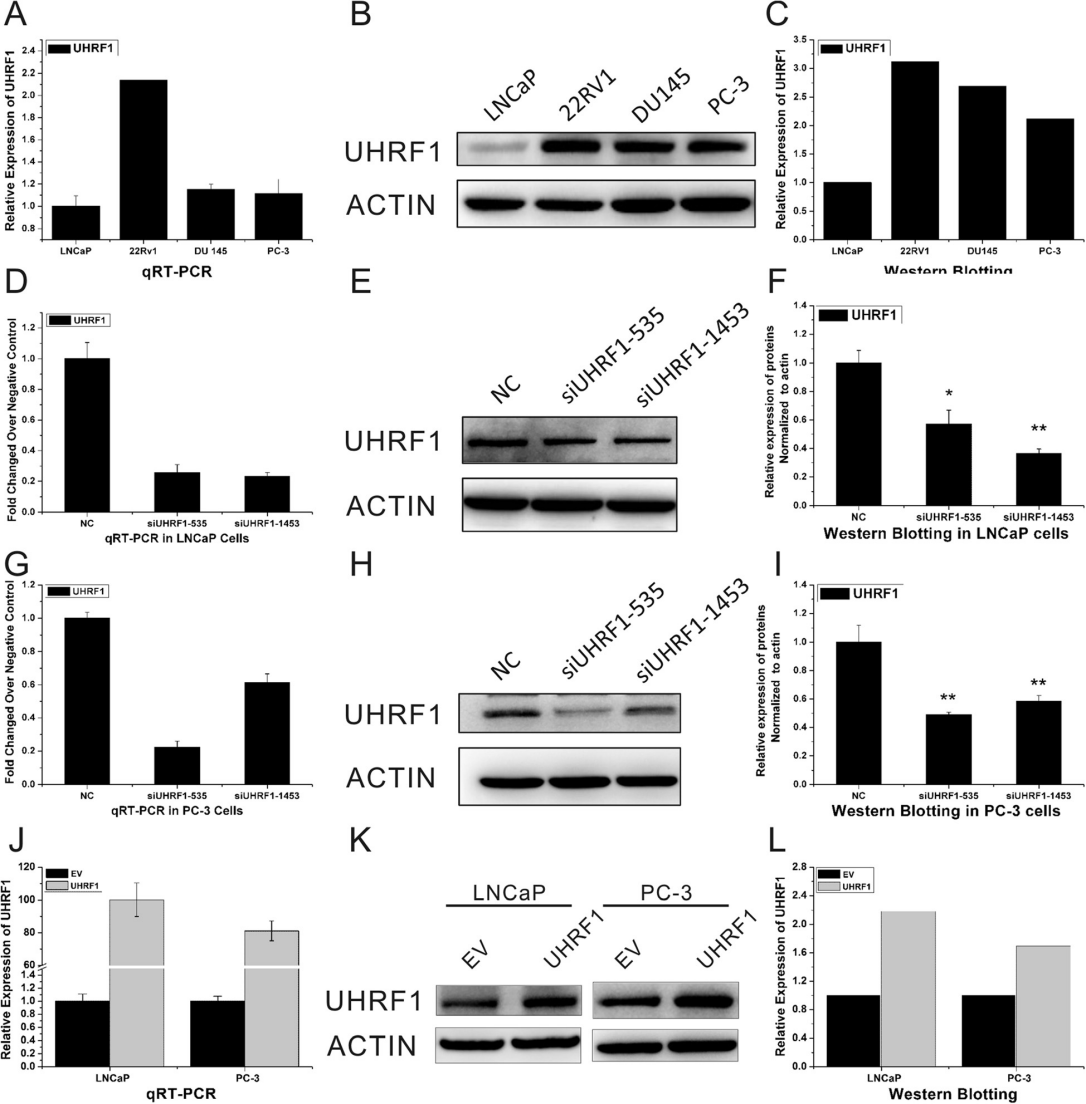
Expression levels of UHRF1 mRNA and protein in PCa cell lines. **a** Expression of UHRF1 mRNA in LNCaP, 22RV1, DU145, and PC-3 cells. **b**, **c** Western blot examination of UHRF1 protein levels in LNCaP, 22RV1, DU145, and PC-3 cells. **d**, **g** Expression of UHRF1 mRNA after transfection with the indicated siRNAs in LNCaP and PC-3cells. **e**, **f** Western blot analysis of UHRF1 protein levels in LNCaP cells 24 h after transfection. **h**, **i** Western blot analysis of UHRF1 protein levels in PC-3 cells 24 h after transfection. **j** Expression of UHRF1 mRNA after transfection with expression plasmid pcDNA 3.1(+)-UHRF1 in LNCaP and PC-3cells. **k**, **l** Western blot analysis of UHRF1 protein levels in PC-3 cells 24 h after transfection. Parts c, f, i and l show the relative grey values of each band (normalized to β-actin). Protein bands from three independent Western blot assays were quantified using Quantity One software (Bio-Rad, USA). Data are reported as the mean ± SD (** *p* < 0.01, Student’s *t*-test)

The mRNA and protein expression levels of UHRF1 were evaluated in the human PCa cell lines 22RV1, DU145, PC-3, and LNCaP by qRT-PCR and Western blot analyses, respectively (Fig. 1a-c). LNCaP is androgen-dependent prostate cancer (ADPCa) cell line, 22Rv1 is weakly androgen-dependent cell line, PC-3 and DU145 are androgen-independent prostate cancer (AIPCa) cell lines. Compared with the less aggressive LNCaP cells, androgen-independent prostate cancer (AIPCa) cell lines DU145 and PC-3 over-expressed UHRF1 in both mRNA and protein levels. Our results were consistent with Babbio’s findings [24]. The finding raised the possibility that the differentially expressed UHRF1 might contribute to the progression of prostate cancer.

To characterize the function of UHRF1 in PCa, UHRF1 siRNA was used to knockdown UHRF1 expression at the mRNA and protein levels. Compared to treatment with the scrambled siRNA control, UHRF1-specific siRNA substantially decreased UHRF1 mRNA and protein levels in LNCaP and PC-3 cells (Fig. 1d-i). Subsequently, we transfected LNCaP and PC-3 cells with the expression plasmid pcDNA 3.1(+) containing the UHRF1 gene. We observed a significant increase of UHRF1 gene expression at both mRNA and protein levels in the transfected cells (Fig. 1j-l).

### UHRF1 influences cell proliferation and cell cycle status in PCa cell lines

**Fig. 2:**
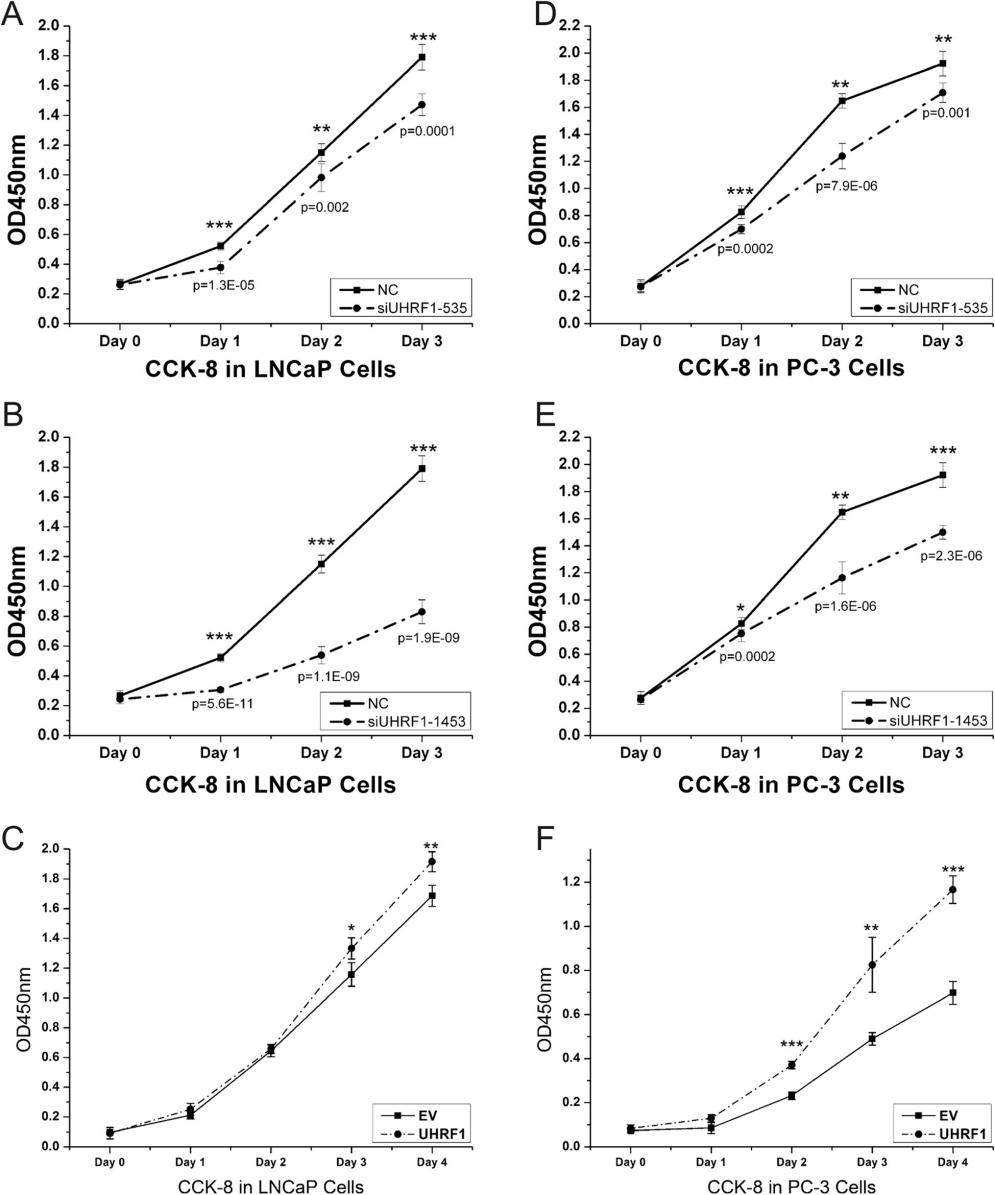
UHRF1 promoted cell proliferation in PCa cells. Cells were seeded into a 96-well plate at 5000 cells/well and examined at 0, 24, 48, and 72 h after transfection. Each experiment was performed in triplicate (*n* = 3). Knockdown of UHRF1 inhibited cell proliferation in LNCaP (**a**, **b**) and PC-3 (**c**, **d**) cells. Overexpression of UHRF1 promoted cell proliferation in LNCaP (**c**) and PC-3 (**f**) cells

We characterized the role of UHRF1 in other cellular processes in PCa cell lines. First, we evaluated the effect of UHRF1 on cell proliferation in LNCaP and PC-3 cells. When cell lines were transfected with siRNA against UHRF1 (siUHRF1), proliferation was decreased compared to cells treated with the nonspecific scrambled siRNA (*p* < 0.001, Fig. 2a, b, d and e). In addition, we further examined the effect of ectopic UHRF1 expression on cell proliferation in LNCaP and PC-3 cells. As shown in Fig. 2, overexpression of UHRF1 significantly promoted proliferation of LNCaP and PC-3 cells compared to negative control cells (Fig. 2c and f).

**Fig. 3:**
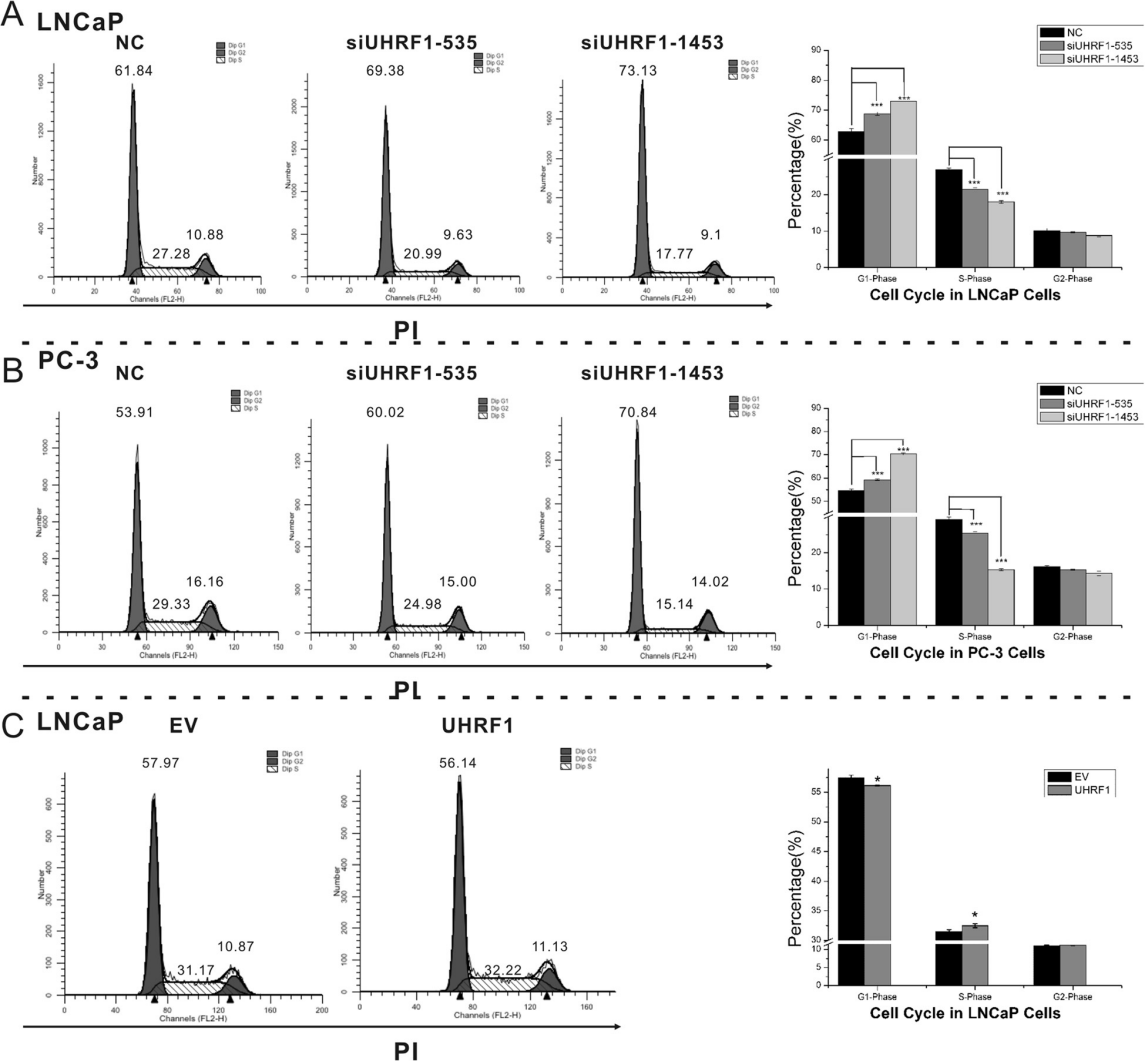
UHRF1 influenced cell cycle status in PCa cells. Cells were transfected with siUHRF1-535, siUHRF1-1453, siNC, pcDNA3.1(+) or pcDNA3.1(+)-UHRF1 plasmid for 48 h, stained with propidium iodide (PI), and used for cell cycle analysis. Treatment with siUHRF1 decreased the percentage of cells in G1 phase and increased the percentage in S phase in LNCaP (**a**) and PC-3 (**b**) cells. Overexpression of UHRF1 induced the decrease in G1 phase and the increase in S phase of LNCaP cells (**c**). Each experiment was performed in triplicate

Given that the cell cycle distribution reflects cell growth, we assessed the influence of UHRF1 on the cell cycle profile of LNCaP and PC-3 cells by flow cytometry. Knockdown of UHRF1 in LNCaP and PC-3 cells increased the percentage of cells in G1 phase and decreased the percentage of cells in S phase compared to treatment with the nonspecific scrambled siRNA (*p* < 0.001; Fig. 3a and b). Furthermore, UHRF1 overexpression slightly induced the decrease in G1 phase and the increase in S phase of LNCaP (*p* < 0.05; Fig. 3c). Taken together, these results indicate that UHRF1 increased the proliferation and growth of LNCaP and PC-3 cells by promoting cell cycle progression.

### UHRF1 suppresses apoptosis of prostate cancer cells

**Fig. 4:**
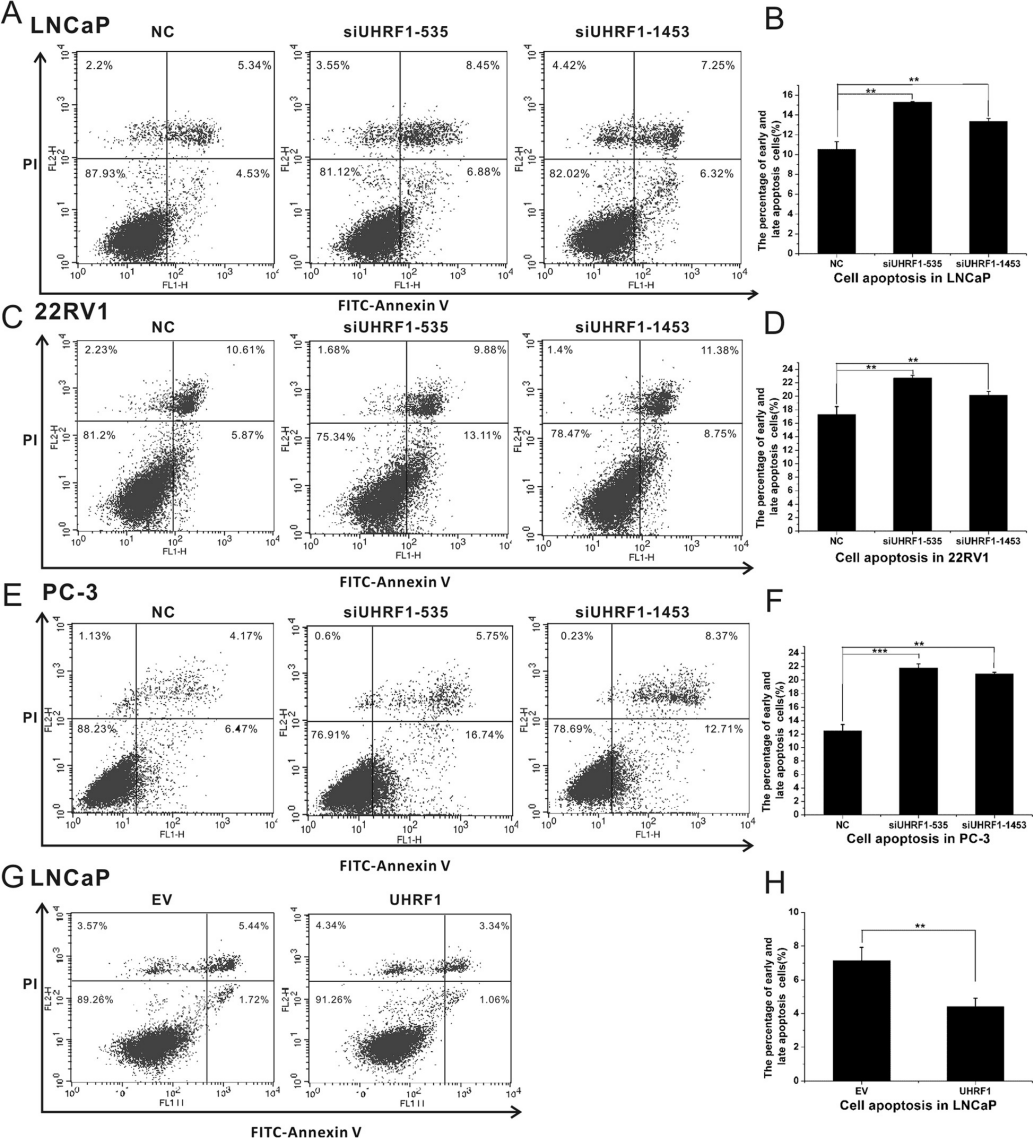
UHRF1 inhibited prostate cancer cell apoptosis. Cells were transfected with siUHRF1-535, siUHRF1-1453, siNC, pcDNA3.1(+) or pcDNA3.1(+)-UHRF1 plasmid for 48 h, and then subjected to cell apoptosis (stained with PI and FITC-Annexin V). Knockdown of UHRF1 caused an increase in early and late apoptotic cells and a decrease in living cells in LNCaP (**a**, **b**), 22RV1 (**c**, **d**) and PC-3 (**e**, **f**). Overexpression of UHRF1 decreased the fraction of apoptotic cells in LNCaP cells (**g**, **h**). Each experiment was performed in triplicate

We next explored the role of UHRF1 in apoptosis in PCa. LNCaP, PC-3 and 22Rv1 cells were treated with siUHRF1 or a scrambled nonspecific siRNA control, stained with Annexin V-FITC/PI, and analysed by flow cytometry. Knockdown of UHRF1 in LNCaP, PC-3 and 22Rv1 cells increased the early and late apoptotic cell fractions (*p* < 0.05; Fig. 4a-c). As expected, we found that overexpression of UHRF1 in LNCaP cells decreased the fraction of apoptotic cells (*p* < 0.01; Fig. 4d).

### Effects of UHRF1 siRNA on the migration of PCa cells in vitro

**Fig. 5:**
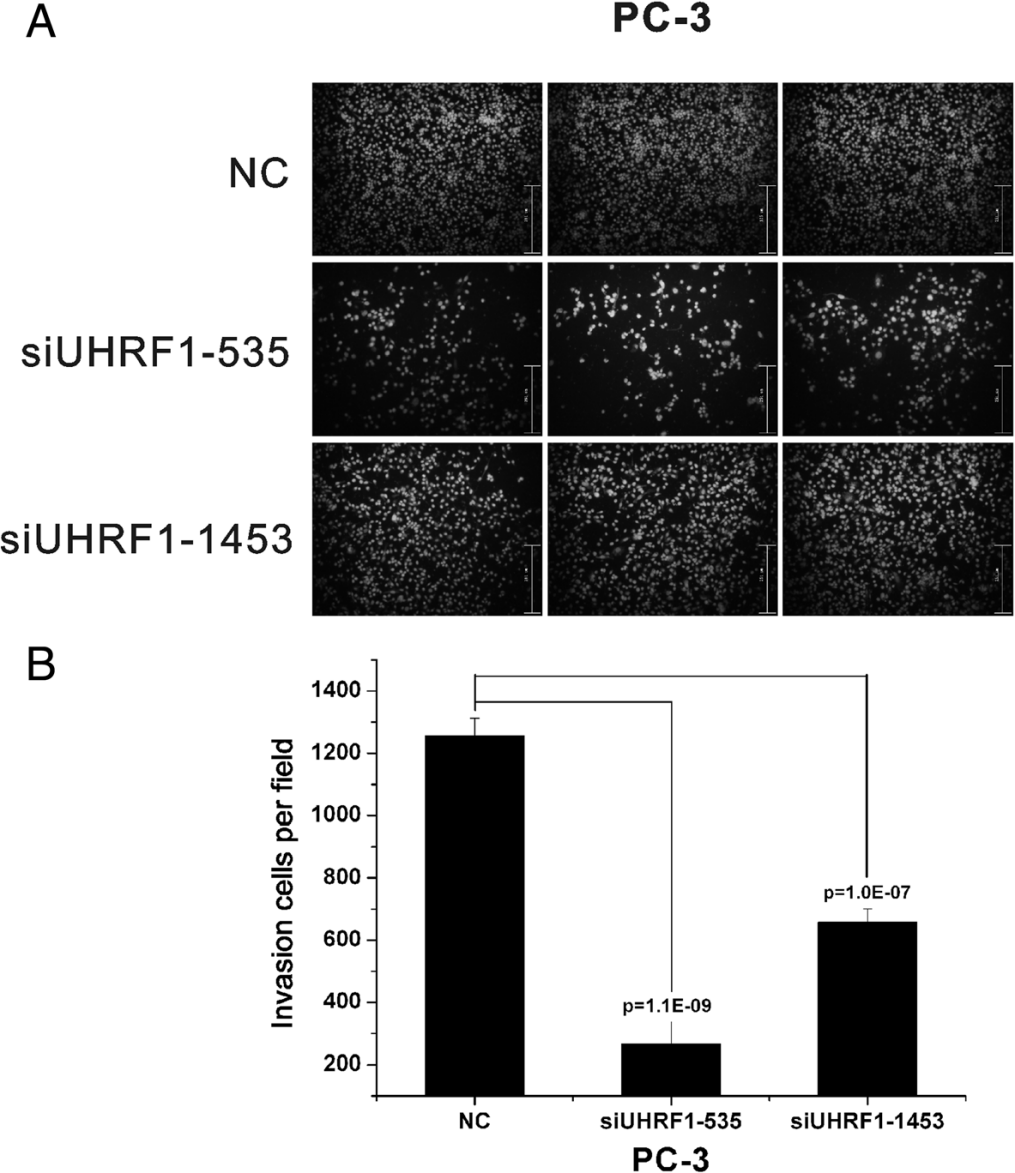
UHRF1 enhanced cell migration. Cells (2 × 10^4^) were transfected with siUHRF1-535, siUHRF1-1453, or scrambled siRNA control and seeded into the upper chamber of a Transwell plate in 0.1 mL media (supplemented with 1 % FBS). At 120 h, cells that had migrated through to the bottom of the Transwell were stained and counted under a reverse microscope. Knockdown of UHRF1 inhibited cell migration in PC-3 cells (**a** and **b**). Each experiment was performed in triplicate (*n* = 3). Bars represent mean ± SD

The migratory ability of PC-3 cells was estimated from the number of cells that migrated through the filter of a Transwell chamber (Fig. 5). The numbers of migrating cells were decreased by 4.737- and 1.91-fold in siUHRF1-535– and siUHRF1-1453–treated PC-3 cells, respectively, compared with the scrambled siRNA-treated control group (*p* < 0.001).

### UHRF1 expression in PCa tissue samples and associations with PCa clinical variables

**Fig. 6:**
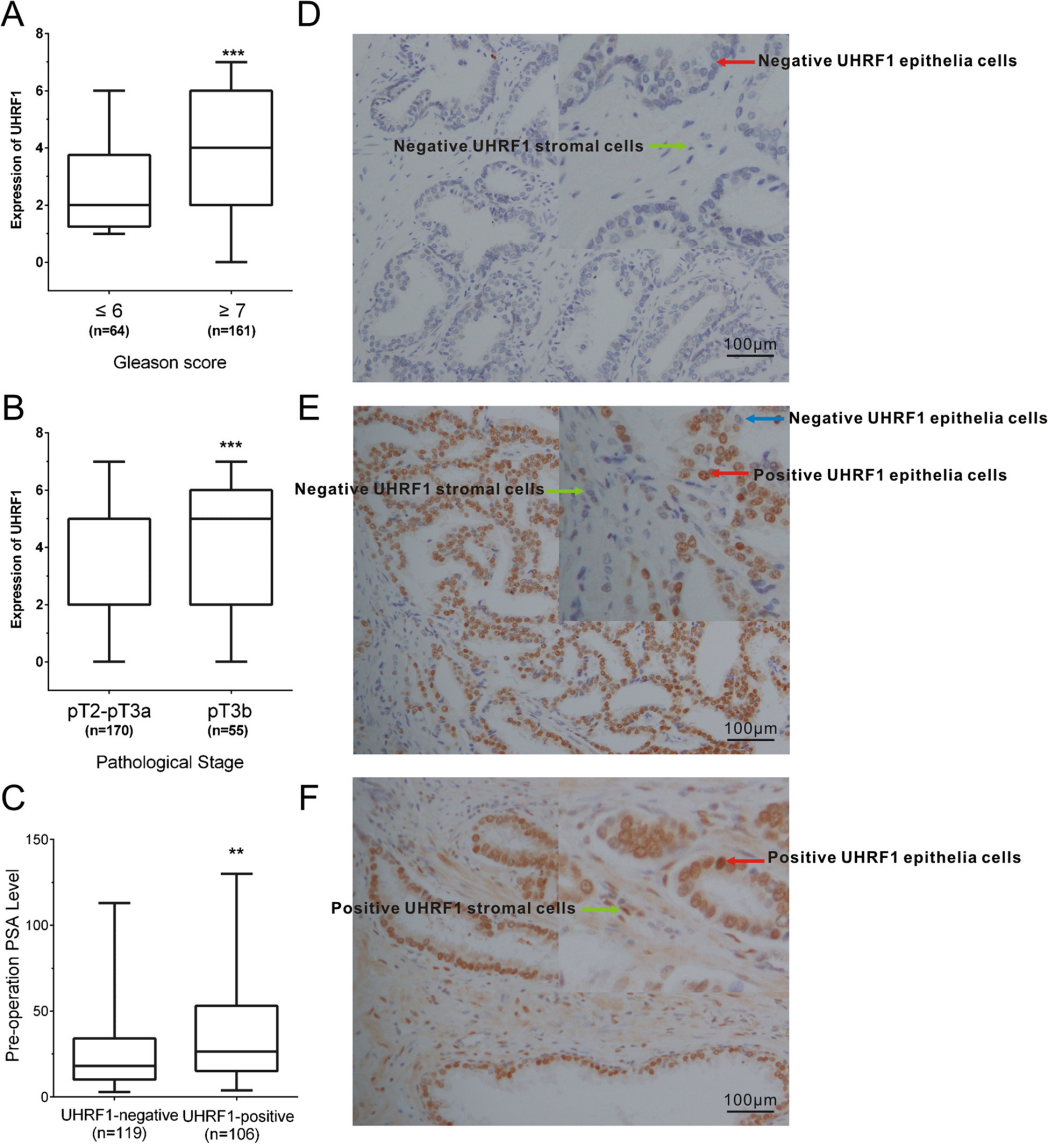
UHRF1 expression in PCa tissue samples and associations with PCa clinical variables. Positive protein expression of UHRF1 was correlated with the Gleason score (**a**), pathological stage (**b**), preoperative PSA level (**c**). **d** UHRF1 staining was negative in normal prostate tissues. **e** Representative positive staining of UHRF1 was mainly detected in epithelia cells on tissue arrays. **f** Representative example of a tumour sample, on which positive staining could be found both in the epithelial cells and stromal tissue. Statistical comparisons between groups of normalized data were performed using Mann–Whitney U-test according to the test condition. Significance was defined as *p* < 0.05 (*, *p* < 0.05; **, *p* < 0.01; ***, *p* < 0.001)

Of the 225 patients with PCa who were included in the study, tissue samples from 106 patients (47.1 %) were positive for UHRF1 protein expression by immunostaining. As shown in Fig. 6, UHRF1 expression was higher in PCa samples compared to PIN samples (2/19; 10.5 %) or normal prostate tissue samples (1/24; 4.0 %; χ^2^ = 24.982, *p* < 0.000; Fig. 6d-f).

Associations between UHRF1 protein expression and the clinicopathologic features of PCa patients after RP are summarized in Table 1. Positive protein expression of UHRF1 was correlated with the Gleason score (Fig. 6a and Table 1), pathological stage (Fig. 6b and Table 1), preoperative PSA level (Fig. 6c and Table 1), and BCR (*p =* 0.000), but was not significantly associated with other clinical characteristics, such as age, LN status, tumour margins, or capsular invasion.

**Table 1:** Correlation between UHRF1 protein expression and clinicopathologic features in PCa patients after RP

Features	UHRF1 protein expression		*P*-Value
	Negative	Positive	
^a^Age at Surgery (*n* = 225)			
Median (Range)	68.00 (65.55-67.81)	67.00 (65.28-67.78)	0.832
Mean	66.68	66.50	
^b^Pathological Stage (pT) (*n*= 225)*			0.028
pT2-pT3a	97	73	
pT3b	22	33	
^c^Lymph Node Status (*n*= 225)			0.143
Positive	7	12	
Negative	112	94	
^c^Tumor Margins (*n*= 225)			0.825
Positive	20	19	
Negative	99	87	
^c^Capsular Invasion (*n*= 225)			0.116
No Invasion	114	96	
Invasion	5	10	
^d^Pre-operation PSA Level (ng/ml) (*n*= 225) **			
Median (Range)	18.00 (21.69, 30.42)	26.47 (31.30, 42.63)	0.002
Mean	26.06	36.97	
^b^Gleason Score (*n* = 225)*			
Gleason≤6	42	22	0.016
Gleason≥7	77	84	
^b^Biochemical Recurrence (*n* = 225)**			
Yes(≥0.2ng/ml)	45	77	<0.000
No(<0.2ng/ml)	74	29	

^a^Mean (t-test)

^b^chi-square test

^c^Fisher’s Exact Test

^d^Mann-Whitney U test

*P<0.05 **P<0.01

### Expression of human UHRF1 protein and BCR of PCa

**Fig. 7:**
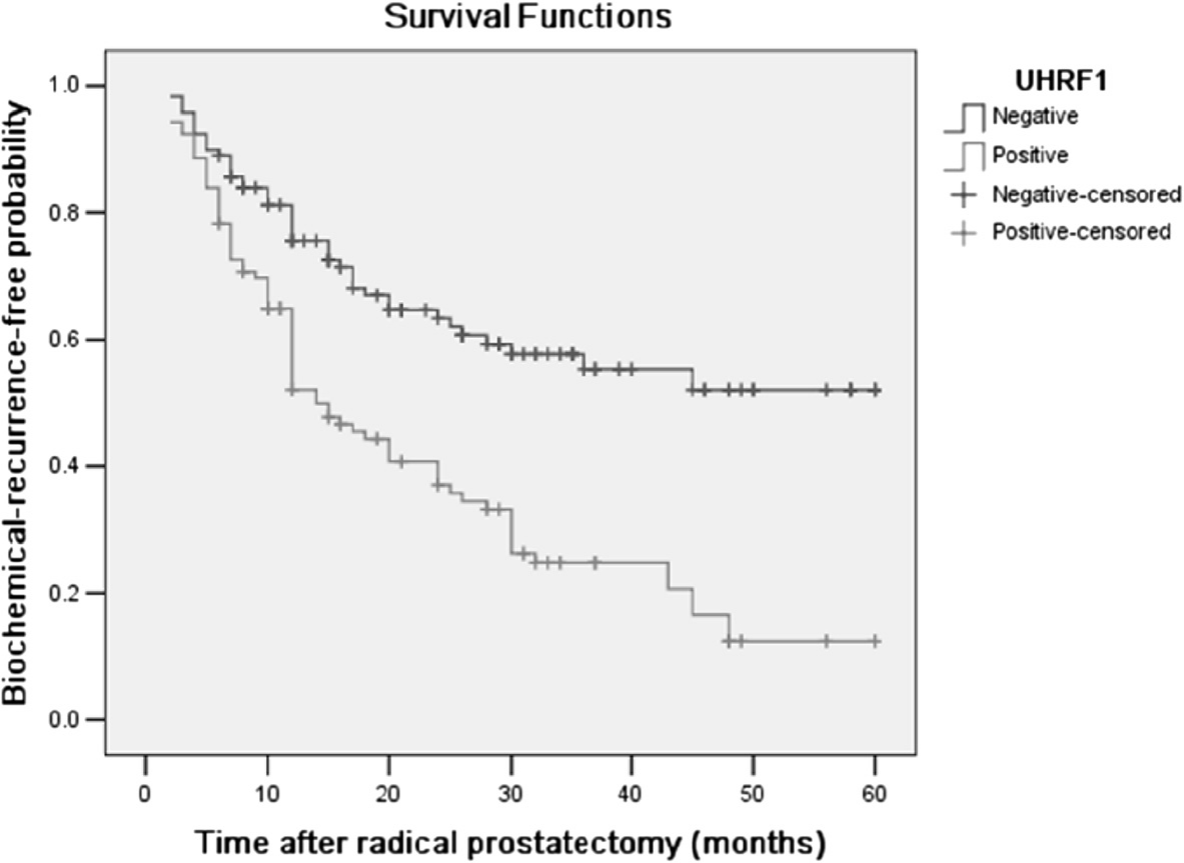
Biochemical recurrence (BCR)-free survival of UHRF1-negative and UHRF1-positive PCa patients. The Kaplan-Meier method was used to draw survival curves for each group of patients based on UHRF1 expression

We evaluated the ability of UHRF1 staining to predict the risk of BCR in patients with PCa who had undergone RP. In PCa specimens, UHRF1 expression was higher in patients who experienced BCR compared to patients who did not (*p* = 0.000; Table 1). To determine whether UHRF1 expression was clinically significant, we assessed the BCR risk in patients who were UHRF1-positive or UHRF1-negative using the Kaplan-Meier method (Fig. 7). The mean BCR-free time and 5-year BCR-free survival rates for the UHRF1-negative and UHRF1-positive PCa patients after RP are shown in Table 2. Comparing UHRF1-negative to UHRF1-positive patients, the 5-year BCR-free survival rates were higher (51.8 vs. 12.4 %, *p* < 0.000) and the mean BCR-free time was longer (38.95 vs. 23.00 months, *p* < 0.000) in UHRF1-negative patients. These results indicate that UHRF1 positivity was correlated with a shorter BCR-free survival time.

**Table 2:** 5-year BCR-free survival rates and median BCR-free time for negative and positive UHRF1 protein expression groups of PCa patients after RP

UHRF1 expression	No. of patients	5-year BCR-free survival rates	BCR-free	
			Mean (months)	95 % CI
Negative	119	51.8 %	38.95	34.2-43.7
Positive	106	12.4 %	23.00	19.1-26.9

*P*<0.001

Finally, we employed a Cox proportional hazards model to assess the independent predictors for BCR-free survival (Table 3). Expression of the UHRF1 protein was an independent prognostic factor of PCa patient outcome after RP. Thus, UHRF1 protein expression level could predict the risk of the BCR after RP.

**Table 3:** Multivariate analysis of BCR-free survival for all PCa patients after RP

Comparison	*P*-Value	Hazard ratio for recurrence	95 % CI
Pre-operation PSA	0.003	1.008	1.003, 1.014
Tumor margins	0.005	0.434	0.242, 0.781
UHRF1 expression	0.005	1.754	1.184, 2.597
Gleason score	0.001	2.366	1.438, 3.892
Pathological Stage	0.035	1.544	1.031, 1.014

## Discussion

UHRF1 is an oncogenic factor that is overexpressed in numerous cancers [25–27]. UHRF1 is able to read both DNA methylation and histone methylation, physically linking these two epigenetic markers, because UHRF1 possesses several domains (e.g., ubiquitin-like domain, PHD finger, SRA domain, and ring finger) [28]. The frequent hypermethylation of promoters of tumour suppressor genes and the overexpression of enzymes that catalyse histone modifications are the two main hallmarks of epigenetic processes in cancer genesis [29, 30]. UHRF1 has been shown to act effectively as a marker to distinguish pancreatic adenocarcinoma, chronic pancreatitis, and normal pancreas tissue [18]. Overexpression of UHRF1 has also been described in breast cancer cells, and could be linked to the degree of breast cancer aggression and its pathological stage [31]. These clinical studies show that incorporating assays for UHRF1 expression could improve the specificity and sensitivity of current cancer diagnostic tests.

In this study, we found that UHRF1 is overexpressed in PCa cells and tissue samples. To explore the potential contribution of UHRF1 to PCa development, we knocked down UHRF1 using siRNA in two PCa cell lines. The siRNAs effectively knocked down UHRF1 expression in PCa cells. UHRF1 knockdown significantly reduced the proliferation and growth of LNCaP and PC-3 cells by repressing cell cycle progression and cell migration (PC-3 cells), but increased the percentage of cells undergoing apoptosis. Moreover, we also found overexpression of UHRF1 significantly promoted cell proliferation and inhibited cell apoptosis of LNCaP. The parts of the results in our study are consistent with Babbio’s findings [24]. Several studies have explored the potential roles of UHRF1 in cancer development. Jenkins *et al.* reported that siRNA-mediated knockdown of UHRF1 significantly inhibited the growth of A549, HeLa, and H1299 cells [32]. Daskalos *et al*. observed reduced cell proliferation and migration properties in lung cancer cells after knocking down UHRF1 [33]. Together with our findings, these observations suggest that increased UHRF1 expression may be involved in PCa carcinogenesis.

The clinical significance of the observed overexpression of UHRF1 in PCa has not been well characterized. We found that overexpression of UHRF1 was significantly correlated with the Gleason score, pathological stage, preoperative PSA level, and BCR, but not with age, LN status, tumour margins, or capsular invasion. Our results indicated a strong correlation between UHRF1 expression and the BCR-free survival of patients. Kaplan-Meier analysis showed that PCa patients with positive UHRF1 expression had a high probability of experiencing BCR after RP compared to UHRF1-negative patients. Cox regression analysis suggested that UHRF1 expression could be a prognostic factor for predicting the risk of BCR.

Despite the combination of increasingly refined surgical techniques and a reduced incidence of surgical complications, the variable disease course in PCa eventually leads to recurrence in about one-third of patients after RP [34]. Distant or local recurrence of PCa does not occur without BCR [35]. Therefore, to achieve the best possibility of long-term disease-free survival for PCa patients after RP, the BCR risk of PCa patients should be assessed. Recent studies have tried to determine tumour cell biological characteristics that are potential prognostic factors. Identification of such factors might help in determining the optimal treatment strategy based on the biology of the individual tumour [36]. Based on our findings, we suggest that PCa patients with low UHRF1 expression should undergo regular monitoring of serum PSA and clinical symptoms. In contrast, PCa patients with high UHRF1 levels could benefit from more extensive monitoring, such as ultrasound-guided biopsy, computed tomography, magnetic resonance imaging, and bone scans.

## Conclusions

In conclusion, UHRF1 expression was upregulated in PCa cell lines and samples. Moreover, UHRF1 knockdown decreased cell proliferation and growth by repressing cell cycle progression and migration, but enhanced apoptosis of PCa cells. Given these results, UHRF1 may be a potential biomarker that can be used as a therapeutic target for PCa. UHRF1 expression in PCa was associated with poorer patient prognosis; therefore, UHRF1 may be a useful prognostic factor for predicting the risk of BCR in PCa patients after RP.
